# Positive association of common variants in CD36 with neovascular age-related macular degeneration

**DOI:** 10.18632/aging.100006

**Published:** 2009-02-12

**Authors:** Naoshi Kondo, Shigeru Honda, Shin-ichi Kuno, Akira Negi

**Affiliations:** ^1^ Department of Surgery, Division of Ophthalmology, Kobe University Graduate School of Medicine, 7-5-2 Kusunoki-cho, Chuo-ku, Kobe 650-0017, Japan; ^2^ Translational Research Informatics Center, Foundation for Biomedical Research and Innovation, 1-5-4 Minatojima-Minamimachi, Chuo-ku, Kobe 650-0047, Japan; ^3^ Clinical Genome Informatics Center, Kobe University Graduate School of Medicine, 7-5-2 Kusunoki-cho, Chuo-ku, Kobe 650-0017, Japan

**Keywords:** age-related macular degeneration, choroidal neovascularization, CD36, genetics, single nucleotide polymorphism, association

## Abstract

Age-related macular degeneration (AMD) is a leading cause of legal blindness among older individuals of industrialized countries. In neovascular AMD, which is an advanced stage of AMD, choroidal neovascularization develops underneath the macula and destroys central vision. Oxidative stress is a hypothesized pathway for the pathophysiology of AMD. CD36 was chosen as a candidate gene for neovascular AMD because the protein plays an important role in this pathway as well as in angiogenesis and in maintaining chorioretinal homeostasis. We tested 19 tag single nucleotide polymorphisms (SNPs) across CD36 for their association with the disease in a Japanese population comprising 109 neovascular AMD subjects and 182 unrelated controls. Five of the 19 SNPs demonstrated a nominally significant association with neovascular AMD (P < 0.05), of which two (rs3173798 and rs3211883) withstood Bonferroni correction for multiple testing (rs3173798, nominal P = 9.96 × 10−4, allele-specific odds ratio = 0.55; rs3211883, nominal P = 2.09 × 10−4, allele-specific odds ratio = 0.50). Population structure analyses excluded stratification artifacts in our study cohort. This study supports the candidacy of CD36 as a novel susceptibility gene for neovascular AMD. Replication of our results in other populations will provide further convincing evidence for the genetic association.

## Introduction

Age-related macular degeneration (AMD) is a leading cause of legal blindness among older individuals of industrialized countries [1]. The advanced stage of AMD is classified into atrophic (dry) or neovascular (wet) types. The atrophic type features a geographic  atrophy of the retinal pigment epithelium (RPE) and photoreceptors of the macula, whereas the neovascular type is characterized by choroidal neovascularization (CNV) and its sequela. Although the growing prevalence of AMD could be attributed to an aging population, the precise etiology remains elusive. Many investigations have established that genetics plays a role in the pathogenesis of AMD. To date, genetic variants in the complement factor H (*CFH*) gene on chromosome 1q32 [2-7] and in two tightly linked genes — age-related maculopathy susceptibility 2 (*ARMS2*), also known as *LOC387715*, and high-temperature requirement factor A1 (*HTRA1*) on 10q26 [8-13] — have demonstrated the strongest replicable associations with AMD across multiple ethnic groups. Variants in two adjacent genes complement factor B, complement component 2 on 6p21 [14,15], and complement component 3 gene on 19p13 [16-18] have also demonstrated replicable associations with AMD among Caucasians.

CD36 is involved in diverse physiological and pathological processes, including scavenger receptor functions (e.g., uptake of oxidized lipids and advanced glycation end products), transforming growth factor-β activation, lipid metabolism, angiogenesis, atherogenesis, and inflammation [19-21]. These wide variety functions are a result of the diverse ligands with which CD36 can interact [19-21]. In particular, CD36 is known as a critical receptor for thrombospondin-1 (TSP-1). The CD36/TSP-1 signal is essential for the inhibition of neovascularization, thereby maintaining the quiescence of the normal vasculature [19,20]. A recent *in vivo* study demonstrated that down-regulation of *CD36* in capillary sprout endothelial cells facilitated angiogenesis and results indicated that the cells were becoming insensitive to antiangiogenic TSP-1 signaling [22]. In the eye, CD36 was reported to play a major role in the inhibition and regression of corneal neovascularization [23]. CD36 also seems to play an important role in maintaining chorioretinal homeostasis. Notably, rats carrying a specific genetic variant of *CD36* have been found to be more susceptible to light-induced retinal damage [24], and are more likely to develop age-related retinal degeneration and chorio-capillary rarefaction [25].

Oxidative stress is widely recognized as an important component in the pathogenesis of AMD [26,27]. The susceptibility of RPE cells to oxidative stress progressively increases with age, and the cumulative oxidative damage causes RPE dysfunction and apoptosis, either directly or through inflammatory processes [26,27]. CD36 could be regarded as a link between oxidative stress and oxidative RPE damage, given that CD36 is involved in the uptake of oxidized lipids by RPE cells [28], which can initiate many of the cellular events relevant to AMD pathogenesis. A recent *in vitro* study reported that the uptake of oxidized low-density lipoprotein (oxLDL) induces the expression of several genes related to oxidative stress, inflammation, and apoptosis for RPE cells [29]. An immuno-histochemical study reported the presence of oxLDL in surgically excised CNV membranes [30]. Furthermore, CD36 is involved in the phagocytosis of photoreceptor outer segments (OSs), where light-induced oxidation of retinal OS phospholipids enhances CD36-mediated phagocytosis [31]. *In vitro* evidence indicates that an exposure of RPE cells to oxLDL compromises the phagocytic ability of RPE cells [32]. This dysfunction can give rise to the accumulation of lipofuscin in RPE cells, which further precipitates oxidative conditions and RPE damage [26,27].

Taken together, CD36 can have specific and important functions in the pathological events involved in AMD and neovascularization. With the hypothesis that genetic variants in *CD36* could be associated with neovascular AMD, we examined the presence of an association of *CD36* variants with the disease.

## Results

### Single-marker associations

The demographic details of the study population are listed in Table 1. Marker information, allelic frequencies, and summary statistics for all evaluated single nucleotide polymorphisms (SNPs) are shown in Table 2. Five of the 19 SNPs showed nominally significant associations with neovascular AMD (*P* < 0.05), of which two (rs3173798 and rs3211883) withstood Bonferroni correction for multiple testing (Bonferroni-corrected *P*= 0.0189 and 0.00397, respectively; Table 2). Applying a permutation procedure for multiple testcorrection also yielded significant *P* values only for thetwo SNPs, rs3173798 and rs3211883 (correctedempirical *P* = 0.0155 and 0.0043, respectively). Theminor allele C at rs3173798 was associated withprotection against neovascular AMD, with a frequency of 0.307 in cases and 0.445 in controls (nominal *P* = 9.96 × 10^−4^; empirical pointwise *P* = 0.0018; per allele odds ratio = 0.55 [95% confidence interval: 0.39-0.79]). The minor allele A at rs3211883 was also protective against the disease, with a frequency of 0.248 in cases and 0.398 in controls (nominal *P* = 2.09 × 10^−4^; empirical pointwise *P* = 5.0 × 10^−4^; per allele odds ratio = 0.50 [95% confidence interval: 0.34-0.72]). Inclusion of age and sex as covariates in logistic regression models did not substantially change the significance of the observed associations (rs3173798, age- and sex-adjusted odds ratio = 0.59 [95% confidence interval = 0.41-0.84], *P* = 3.10 × 10^−3^, additive model; rs3211883, age- and sex-adjusted odds ratio = 0.5 [95% confidence interval = 0.36 - 0.77],  *P* =  7.0 × 10^−4^,  additive  the model). The two SNPs, rs3173798 and rs3211883, were highly correlated with each other (r^2^ = 0.80); thus, their effects could not be separated statistically (fitting one in conditional logistic regression model rendered the other redundant). When either SNP rs3173798 or rs3211883 was fitted in the logistic regression, all other SNPs showing nominally significant association (rs10499862, rs3173800, and rs17154232) were redundant.

**Table 1.** Characteristics of the study population.

	**Neovascular AMD**	**Controls**
Number of subjects	109	182
Gender (male/female)	87/22	110/72
Mean age ± SD (years)	76 ± 7.3	72 ± 5.8
Age range (years)	57-91	56-95

**Table 2.** Results of single-marker association test.

			Minor Allele Frequency		Association Results			
SNP	Location	Minor Allele	Cases	Controls	Allelic *P*-value (Empirical Pointwise *P*-value)*	Allelic OR (95% CI)	Corrected Empirical *P*-value†	Bonferroni Corrected *P*-value‡
rs12531609	Intron 1	T	0.165	0.223	0.0945 (0.138)	0.69 (0.45-1.07)	0.608	1
rs3211816	Intron 3	A	0.509	0.475	0.428 (0.451)	1.15 (0.82-1.60)	0.995	1
rs10499862	Intron 3	C	0.106	0.187	0.00895 (0.0126)	0.51 (0.31-0.85)	0.113	0.17
rs3211849	Intron 3	A	0.289	0.269	0.606 (0.628)	1.10 (0.76-1.60)	1	1
rs3211851	Intron 3	C	0.202	0.253	0.160 (0.20)	0.75 (0.50-1.12)	0.799	1
rs1054516	Intron 3	C	0.395	0.459	0.130 (0.136)	0.77 (0.55-1.08)	0.726	1
rs3173798	Intron 3	C	0.307	0.445	9.96 × 10^−4^ (0.0018)	0.55 (0.39-0.79)	0.0155	0.0189
rs3211870	Intron 4	C	0.454	0.511	0.184 (0.181)	0.80 (0.57-1.12)	0.839	1
rs1358337	Intron 4	G	0.349	0.319	0.457 (0.454)	1.14 (0.80-1.63)	0.996	1
rs3211883	Intron 4	A	0.248	0.398	2.09 × 10^−4^ (5.0 × 10^−4^)	0.50 (0.34-0.72)	0.0043	0.00397
rs3173800	Intron 4	T	0.404	0.289	0.00427 (0.00570)	1.67 (1.17-2.38)	0.0538	0.0812
rs1924	Intron 5	A	0.161	0.220	0.0824 (0.0877)	0.68 (0.44-1.05)	0.570	1
rs17154232	Intron 6	C	0.087	0.151	0.0250 (0.0411)	0.54 (0.31-0.93)	0.256	0.475
rs17154233	Intron 6	C	0.266	0.203	0.0801 (0.0776)	1.42 (0.96-2.11)	0.555	1
rs3211908	Intron 7	T	0.142	0.146	0.91 (1)	0.97 (0.60-1.57)	1	1
rs17154258	Intron 8	G	0.142	0.184	0.191 (0.218)	0.73 (0.46-1.17)	0.860	1
rs1527483	Intron 11	A	0.179	0.176	0.925 (1)	1.02 (0.66-1.58)	1	1
rs3211958	Intron 14	G	0.367	0.396	s0.492 (0.531)	0.89 (0.63-1.25)	0.998	1
rs7755	3′UTR	G	0.491	0.420	0.0978 (0.124)	1.33 (0.95-1.86)	0.631	1

### Haplotype analysis

**Figure 1.**
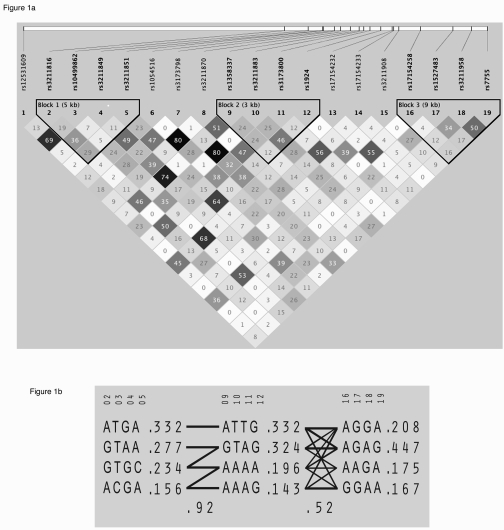
Linkage disequilibrium structure and haplo-typic architecture in *CD36.* (**A**) Haploview plot defining haplotype block structure of the *CD36* region. Linkage disequilibrium (LD) was measured using data from all subjects in the present study. The haplotype blocks were determined using the criteria described by Gabriel et al. [33]. The physical position of each SNP is presented in the upper diagram. Each box provides estimated statistics of the coefficient of determination (r^2^), with darker shades representing stronger LD. (**B**) Haplotypes in the haplotype blocks across the *CD36* region. There are three haplotype blocks across the region. The haplotype frequencies are shown to the right of each haplotype. Only haplotypes having a frequency greater than 1% are shown. The SNP numbers across the top of the haplotypes correspond to those in the Haploview plot. A multiallelic D′ statistic, which indicates the level of recombination between two blocks, is shown in the crossing area. Connections from one block to the next were shown for haplotypes of greater than 10% frequency with thick lines and greater than 1% frequency with thin lines.

The pairwise linkage disequilibrium (LD) structure was constructed with all SNPs evaluated (Figure 1a). Using the criteria described by Gabriel et al. [33], three haplotype blocks were defined (Figure 1a). Haplotype analyses from all blocks revealed that the association with neovascular AMD was restricted to block 1 and 2, as demonstrated by the significant omnibus results (omnibus *P* = 0.00482 and 0.00181, respectively; Table 3). As shown in Table 3, one haplotype in block 1 and two haplotypes in block 2 were found to be significantly associated with the disease after correction for multiple testing (permutation *P* < 0.05). A risk haplotype (underlined in Table 3) showed a solid spine of LD across blocks 1 and 2, with haplotype frequencies of 0.404 in affected individuals and 0.288 in controls (*P* = 0.0043; odds ratio = 1.67 [95% confidence interval = 1.17-2.38]; Figure 1b). This haplotype was completely described by the allele T at rs3173800. The protective allele A at rs3211883 was split into two different haplotypes, one of which showed statistical significance for protection against neovascular AMD (*P *= 0.0067; odds ratio = 0.48 [95% confidence interval = 0.28-0.83]; Table 3).

**Table 3.** Association of *CD36* haplotype blocks with neovascular AMD. Associations of 3 haplotypes, ATGA in block 1 and ATTG and AAAG in block 2, remained statistically significant after correction for multiple testing (permutation *P* = 0.0325, 0.0325, and 0.0453, respectively). The evidence for association of haplotype ACGA in block 1 disappeared after correction for multiple testing (permutation *P* = 0.0622). The risk haplotype showing a solid spine of LD across blocks 1 and 2 was underlined.

		Frequency				
	Haplotype*	Cases	Controls	*P*-value†	OR (95% CI)	Omnibus *P*-value‡
Block 1	ATGA	0.404	0.288	0.0043	1.67 (1.17-2.38)	0.00482
	GTAA	0.289	0.269	0.606	1.10 (0.76-1.60)	
	GTGC	0.202	0.253	0.160	0.75 (0.50-1.12)	
	ACGA	0.106	0.187	0.0089	0.51 (0.31-0.85)	
Block 2						
	ATTG	0.404	0.288	0.0043	1.67 (1.17-2.38)	0.00181
	GTAG	0.344	0.313	0.443	1.15 (0.80-1.64)	
	AAAA	0.156	0.219	0.06	0.65 (0.42-1.02)	
	AAAG	0.092	0.173	0.0067	0.48 (0.28-0.83)	
Block 3						
	AGAG	0.491	0.420	0.0978	1.33 (0.95-1.86)	0.328
	AGGA	0.188	0.220	0.362	0.82 (0.54-1.25)	
	AAGA	0.179	0.173	0.858	1.04 (0.67-1.62)	
	GGAA	0.142	0.181	0.220	0.75 (0.47-1.19)	

### Assessment of population stratification

Hidden population stratification between cases and controls can generate a false positive association. The population stratification was examined by STRUCTURE [34] using 26 unlinked genome-wide SNPs. We found no evidence of significant stratification in our study cohort [*Pr* (*K* = 1 > 0.99)], indicating that population stratification did not account for association signals detected in the present study.

## Discussion

We tested biological candidate gene *CD36* and found that common variants in this gene are associated with neovascular AMD in a Japanese population. We confirmed the lack of population stratification between case and control subjects in the present study. Our results implicate *CD36* as a previously unknown genetic risk factor for neovascular AMD.

We identified two protective variants satisfying stringent statistical thresholds for significance; rs3211883 was the most significant SNP (nominal allelic *P* = 2.09 × 10^−4^), followed by rs3173798 (nominal allelic *P* = 9.96 × 10^−4^). The two SNPs were highly correlated with each other (r^2^ = 0.80); therefore, their effects could not be separated statistically in our dataset. The biological basis of the associations is currently unknown because the two SNPs do not reside in the coding sequence of *CD36*. Using the FASTSNP program [35],  we predicted binding of the CDX1 intronic enhancer to the sequence containing rs3211883 and rs3173798 to be located in a potential splice site. Thus, these two SNPs could have non-coding effects on gene function; however, exhaustive resequencing of the locus is required to search potentially undiscovered and more important causative variants.

*CD36* is located on chromosome 7q11.2, a region that has not been previously implicated in AMD. We examined SNPs across a 366 kb region harboring *CD36* and two flanking genes  (*GNAT3* and *SEMA3C*) in  an available database, the NEI/NCBI dbGAP database (http://www.ncbi.nlm.nih.gov/projects/gap/cgi-bin/study.cgi?id=phs000001). This database provides results of a genome-wide association (GWA) analysis between 395 individuals with AMD and 198 controls from the National Eye Institute Age-Related Eye Disease Study (AREDS). This analysis did not include the two most significant SNPs (rs3173798 and rs3211883) or any of the three SNPs (rs10499862, rs3173800, and rs17154232) that showed nominally significant associations in our study. The GWA study looked at five *CD36* SNPs (rs1194182, rs3211822, rs3211885, rs1405747, and rs7755) and found no significant association (all nominal *P* > 0.05). Of the five GWA SNPs, rs7755 was also typed in our study and all of the remaining GWA SNPs were captured by our tag SNPs; rs1194182 was highly correlated with rs3211849 (r^2^ = 0.95) and rs3211822, rs3211885, and rs1405747 were a  perfect proxy (r^2^ =1) for rs3211816, rs3211870, and rs7755, respectively, according to the HapMap JPT data. Consistent with the GWA data from AREDS, none of the four proxy SNPs (rs3211849, rs3211816, rs3211870, and rs7755) showed a significant association with neovascular AMD in our study (all nominal *P* > 0.05), indicating that the GWA study in the AREDS cohort was unable to capture the genetic effects detected in the present study.

Cumulative oxidative stress is an important component of AMD pathogenesis because of its contribution to RPE damage and subsequent pathology such as the activation of inflammatory responses in the Bruch membrane and choroids [26,27]. It is possible that altered CD36 biologic behavior, which is defined by common variations in this gene, could be a contributing factor for this pathogenic sequence of events given its ability to scavenge oxidized lipids [28,29]  and  phagocytose OSs under conditions of increased oxidative stress [31]. The ability of CD36 to mediate the antiangiogenic activity of TSP-1 would also predict a proangiogenic consequence of CD36 dysfunction [19,20]. Further support for the involvement of *CD36* in AMD pathogenesis can be found in studies involving CD36-deficient animals. Rats carrying a specific genetic variant of *CD36* have been shown to be more susceptible to light-induced retinal damage [24], and are more likely to develop an age-related retinal degeneration and choriocapillary rarefaction [25]. This observation could serve as a link between the genetic association  observed  in  our  study and prior evidence that a markedly decreased choroidal circulation precedes the appearance of CNV in neovascular AMD [36].

In conclusion, we report a novel association between common variants in *CD36* and neovascular AMD in a Japanese population. Although the underlying causative biological perturbation related to these variants is not yet clear, this study supports the candidacy of *CD36* as a novel susceptibility gene for neovascular AMD. Replication of our results in other populations will provide further convincing evidence for the association of *CD36* variants with neovascular AMD.

## Methods

Study participants. This study was approved by the Institutional Review Board at Kobe University Graduate School of Medicine and was conducted in accordance with the Declaration of Helsinki. Written informed consent was obtained from all subjects. All cases and controls included in this study were Japanese individuals recruited from the Department of Ophthalmology at Kobe University Hospital in Kobe, Japan.

All patients with neovascular AMD received ophthalmic examinations, including visual acuity measurement, slit-lamp biomicroscopy of the fundi, color fundus photographs, optical coherence tomography, fluorescein angiography, and indocyanine green angiography. All of our study subjects with neovascular AMD had CNV and associated manifestations such as nondrusenoid pigment epithelial detachment, serous or hemorrhagic retinal detachments, subretinal or sub-RPE hemorrhages, and fibrosis, and thus, were categorized as having clinical age-related maculopathy staging system (CARMS) stage 5 [37]. Patients with polypoidal choroidal vasculopathy and secondary choroidal neovascular diseases such as degenerative myopia, ocular trauma, angioid streaks, idiopathic CNV, and presumed ocular histoplasmosis were excluded from our study. The control subjects were 56 years of age or older and were defined as individuals without macular degeneration and macular changes such as drusen or pigment abnormalities. Thus, controls were categorized as having CARMS stage 1 [37] on the basis of comprehensive ophthalmic examinations.

SNP selection. To comprehensively yet efficiently screen *CD36* sequences for genetic variations in a Japanese population, we ran Tagger tool [38] from HapMap Project database for the Japanese in Tokyo (JPT) population [39] (minor allele frequency cutoff was set at 0.1; r^2^ cutoff was set at 0.8; and the Tagger Pairwise mode was used). Nineteen tag SNPs across a 74.5 kb region encompassing *CD36* were selected for genotyping. Based on the HapMap JPT data, these 19 SNPs captured 121 of 123 SNPs in *CD36* exhibiting a minor allele frequency greater than 10% with a mean r^2^ value of 0.97. Thus, our set of 19 SNPs is highly representative of the common genetic variation in *CD36* because it acts as a proxy marker for other untyped SNPs in this region.

Genotyping. Genomic DNA was extracted from the peripheral blood using a standard methodology. Genotyping was performed using TaqMan^®^ SNP Genotyping Assays (Applied Biosystems, Foster City, CA) on a StepOnePlus™ Real-Time PCR System (Applied Biosystems) in accordance with the supplier's recommendations.

Statistical analysis. Each marker was tested for association using a software package, PLINK v1.00 (http://pngu.mgh.harvard.edu/purcell/plink/) [40]. In addition to obtaining nominal *P*-values, empirical *P*-values were generated by 10,000 permutation tests using Max (T) permutation procedure implemented in PLINK [40]. In this procedure, two sets of empirical significance values were calculated: pointwise estimates of an individual SNP's significance (empirical pointwise *P*-values) and corrected values for multiple testing (corrected empirical *P*-values). We also applied a Bonferroni correction [41], which is the most conservative correction for multiple testing, where nominal *P*-values were multiplied by 19 (the number of SNPs tested for association). To adjust for age and sex differences between the case and control subjects, logistic regression analyses were performed using SNPStats (http://bioinfo.iconcologia.net/SNPstats), with age and sex controlled as covariates. Age and sex were included in this model as a continuous covariate measured in years and a categorical covariate, respectively. Deviations from Hardy-Weinberg equilibrium were tested using the chi-square test (1 degree of freedom), and all of the 19 SNPs passed the Hardy-Weinberg equilibrium tests in both the case and control subjects (*P* > 0.001) [41]. To dissect multiple association signals due to LD patterns, we conducted conditional logistic regression analysesusing the logistic and condition options in PLINK. The FASTSNP program  (http://fastsnp.ibms.sinica.edu.tw/pages/input_CandidateGeneSearch.jsp) was used to investigate the potential function of SNP [35].

A software package, Haploview, was used for assessing LD patterns and haplotype association statistics [42]. Haplotype blocks were determined using the algorithm of Gabriel et al [33]. To correct for multiple testing in the haplotype analysis, 10,000 permutations were run using Haploview. Odds ratios and 95% confidence intervals for haplotype-specific risks were calculated using VassarStats (http://faculty.vassar.edu/lowry/VassarStats.html). An omnibus (or global) test of the haplotype association was conducted with PLINK.

Population stratification errors are a major problem in case-control studies because they can generate spurious positive associations [41]. The population stratification should be minimized in our study cohort given the genetic homogeneity of the Japanese population. However, to exclude a potential stratification in our study cohort, we examined the population stratification by a software package, STRUCTURE [34], as performed in previous genetic association studies on Japanese populations [43-45]. The following 26 polymorphic SNPs, which were randomly distributed along the genome and are not in LD with each other (r^2^ < 0.035), were used for this analysis: rs3818729 (1p13.2), rs13388696 (2p23.1), rs2305619 (3q25.32), rs6876885 (5p15.1), rs6459193 (6p11.2), rs3779109 (7p22.1), rs6468284 (8p12), rs955220 (9p24.3), rs4838590 (10q11.22), rs12806 (10q24.2), rs2019938 (11p15.5), rs609017 (11q24.3), rs3912640 (12p13.2), rs2283299 (12p13.33), rs715948 (12q13.3), rs7328193 (13q12.11), rs1048990 (14q13.2), rs16948719 (15q22.31), rs11076720 (16q24.3), rs1051009 (17p13.2), rs1292033 (17q23.1), rs7239116 (18q11.2), rs892115 (19p13.2), rs844906 (20p11.21), rs2825761 (21q21.1), and rs3884935 (22q13.1). The log likelihood of each analysis at varying number of *K* (the number of populations) was estimated from three independent runs (20,000 burn in and 30,000 iterations). The best estimate of *K* was identified by computing posterior probabilities* Pr* (*K* = 1, 2, 3, 4, or 5) based on the log likelihood as described by Pritchard et al. [46].
